# The role of optical coherence tomography angiography in distinguishing ischemic versus non-ischemic central retinal vein occlusion

**DOI:** 10.1186/s12886-022-02637-y

**Published:** 2022-10-28

**Authors:** Weiting An, Qi Zhao, Rongguo Yu, Jindong Han

**Affiliations:** Tianjin Key Laboratory of Retinal Functions and Diseases, Tianjin Branch of National Clinical Research Center for Ocular Disease, Eye Institute, School of Optometry, Tianjin Medical University Eye Hospital, 300384 Tianjin, China

**Keywords:** Central retinal vein occlusion, Optical coherence tomography angiography, Ischemic, Non-ischemic

## Abstract

**Introduction:**

To observe macular microvascular changes in patients with ischemic and non-ischemic central retinal vein occlusion (CRVO) by optical coherence tomography angiography (OCTA), and explore the value of OCTA in differentiating ischemic and non-ischemic CRVO.

**Methods:**

Cross sectional study. Fifty patients diagnosed as CRVO with macular edema were included. Macular edema in all patients were regressive after three consecutive anti-VEGF treatment. Patients were divided into ischemic and non-ischemic group according to ultra-wide-angle fundus fluorescein angiography (UWFFA). All patients underwent BCVA, IOP, color fundus photography, UWFFA and OCTA. The following parameters were measured: (1) Vessel density (VD): superficial and deep whole VD (SVD, DVD), superficial and deep central fovea VD (SFVD, DFVD), superficial and deep parafoveal VD (SPFVD, DPFVD); (2) Central foveal retinal thickness (CRT); (3) Area of foveal avascular zone (FAZ), perimeter of FAZ (PERIM), avascular index of FAZ (AI) and VD within a width of 300 microns around the FAZ region (FD-300). Comparison between ischemic and non-ischemic group was performed by two independent sample t-tests. Receiver operating characteristic (ROC) curve analysis was used to measure the area under the curve (AUC) of VD for predicting ischemic CRVO.

**Results:**

There were no significant differences in IOP, SFVD, DFVD and CRT between ischemic and non-ischemic group, and significant differences in age, BCVA, SVD, SPFVD, DVD, DPFVD, FAZ area, PERIM, AI and FD-300 between ischemic and non-ischemic group. ROC curve analysis showed AUC of DVD and DPFVD in predicting ischemic CRVO was highest (0.962). the threshold was 38.40%, and the sensitivity was 100%, but the specificity of DVD (92.3%) was significantly higher than that of DPFVD (84.6%). Therefore, DVD ≤ 38.40% can be used as the best threshold for determining ischemic CRVO.

**Conclusion:**

OCTA can quantitatively evaluate the macular microvascular structure of CRVO, which is helpful to distinguish ischemic from non-ischemic CRVO.

## Introduction

Central retinal vein occlusion (CRVO) is the second most common retinal vascular disorder next to diabetic retinopathy. It can be divided into ischemic and non-ischemic CRVO according to the non-perfusion (NP) area of ​​retinal capillaries caused by obstruction. Retinal ischemia is the main complication of CRVO and may result in macular ischemia which limits visual recovery and/or in anterior segment neovascularization and neovascular glaucoma (NVG) [1]. NVG develops in at least 23% of the eyes after 15 months in ischemic CRVO, however, development of NVG is rare in non-ischemic CRVO [2]. According to the previous research, Visual acuity [3], RAPD [4], b-wave amplitude on ERG [5], Goldmann perimetry and FFA can be used to distinguish CRVO ischemia classification. the retinal capillary non-perfusion area (NPA) measured by FFA is the most commonly used parameter to distinguish ischemic from non-ischemic RVO [6–8]. In recent years, the application of ultra-wide-angle FFA (UWFFA) can widely present NP area, neovascularization and vascular leakage in peripheral retina [9]. However, it can only be used to observe superficial capillary plexus (SCP), but cannot be used to observe another capillary plexus, especially the deep capillary plexus (DCP) [10]. In addition, the assessment of capillary alternations by FFA is limited in acute phase of CRVO because of edema and hemorrhage [11]. In addition, Macular fovea is the area responsible for the central vision. It is essential when evaluating CRVO that the perfusion status of the macula as well as whether or not there is preservation or destruction of the perifoveal capillaries is determined, in addition to the evaluation of the presence/absence and extension of ischemia in the midperipheral and peripheral retina. Optical coherence tomography (OCTA) is a new non-invasive, high-resolution, and layer-specific blood flow imaging technology providing microvascular assessment by using blood cell movement as natural contrast [12]. It can automatically measure the vessel density (VD) in the SCP and DCP and the area of foveal avascular zone (FAZ). In light of the above information, we aimed to observe the difference of macular microvascular structure in patients with ischemic and non-ischemic CRVO by OCTA, and to explore the value of OCTA in differentiating ischemic and non-ischemic CRVO.

## Methods

A cross sectional study. This study adhered to the tenets of the Helsinki Declaration and was approved by ethics committee of Tianjin Medical University Eye Hospital. Written informed consent was obtained from all patients.

Fifty patients (50 eyes) diagnosed as CRVO with macular edema were included in this study in Tianjin Medical University Eye Hospital from January 2020 to March 2021. There were 24 males (24 eyes) and 26 females (26 eyes). Inclusion criteria: (1) The age is over 18 years old; (2) Patients were diagnosed as macular edema secondary to CRVO [6]; (3) Macular edema in all patients were regressive because of anti-VEGF treatment for 3 consecutive times. Exclusion criteria: (1) Patients with other ophthalmic diseases (diabetic retinopathy, retinal artery occlusion, age-related macular degeneration, etc.); (2) Patients with significant media opacity affected the fundus imaging; (3) Patients with history of retinal laser photocoagulation; (4) Image with low quality (signal strength index < 6/10). Ischemic CRVO was defined by the Central Vein Occlusion Study (CVOS) group by the presence of ≥ 30 DA of retinal capillary non-perfusion on ultra-wide-angle fundus fluorescein angiography (UWFFA) [6]. The CRVO subtypes (ischemic and non-ischemic) were determined by a retinal specialist in a blind manner to prevent observation bias. Patients were divided into ischemic (26 eyes) and non-ischemic group (24 eyes) according to UWFFA.

All patients underwent a comprehensive ophthalmic examination, including best-corrected visual acuity (BCVA), intraocular pressure (IOP) measurement, slit-lamp examination, Widefield color fundus photography, UWFFA and OCTA. The examination was performed independently by the same skilled person to avoid measurement bias. The scanning range of OCTA was 3 mm×3 mm in macular area. The images were analyzed at SCP (by automated segmentation selecting area between internal limiting membrane and external boundary of ganglion cell layer) and DCP (by automated seg-mentation selecting area between inner plexiform layer and outer plexiform layer) [13]. Some parameters as followed were measured by the built-in software. (1) VD: the superficial and deep whole vessel density (SVD, DVD), the superficial and deep central fovea vessel density (SFVD, DFVD), the superficial and deep parafoveal vessel density (SPFVD, DPFVD); (2) Central foveal retinal thickness (CRT); (3) FAZ area, perimeter of FAZ (PERIM), avascular index of FAZ (AI), VD within a width of 300 microns around the FAZ region (FD-300). The AI was defined as the ratio between the measured perimeter and a perimeter with the same size circular area, with a perfectly circular FAZ having an AI equal to 1.

Analyses were performed using SPSS 26.0. All visual acuity data were converted to the logarithm of minimal angle of resolution (LogMAR) scale for analyses. All quantitative data that were normally distributed were expressed as Mean ± SD. Comparison between the ischemic and non-ischemic group was performed by two independent sample *t*-tests, *P* < 0.05 was considered statistically significant. Receiver operating characteristic (ROC) curve analysis was used to measure the area under the curve (AUC) of VD for predicting ischemic CRVO. The threshold and corresponding sensitivity and specificity were determined. AUC > 0.9 was considered as a good prediction efficiency.

## Results

The age of patients in the ischemic and non-ischemic group was 66.15 ± 8.66 and 60.17 ± 10.18 years old, respectively, and the difference between the two groups was statistically significant (*t* = 2.231, *P* = 0.031). The LogMAR BCVA of patients was 1.00 ± 0.84 and 0.32 ± 0.16 in the ischemic and non-ischemic group, respectively, and there was significant difference between the two groups (*t* = 3.856, *P* ≤ 0.001). The IOP of patients in the ischemic and non-ischemic group was 13.65 ± 3.60mmHg and 14.67 ± 3.10mmHg, respectively, and the difference between the two groups was not statistically significant (t=-1.063, P = 0.293).

**Fig. 1:**
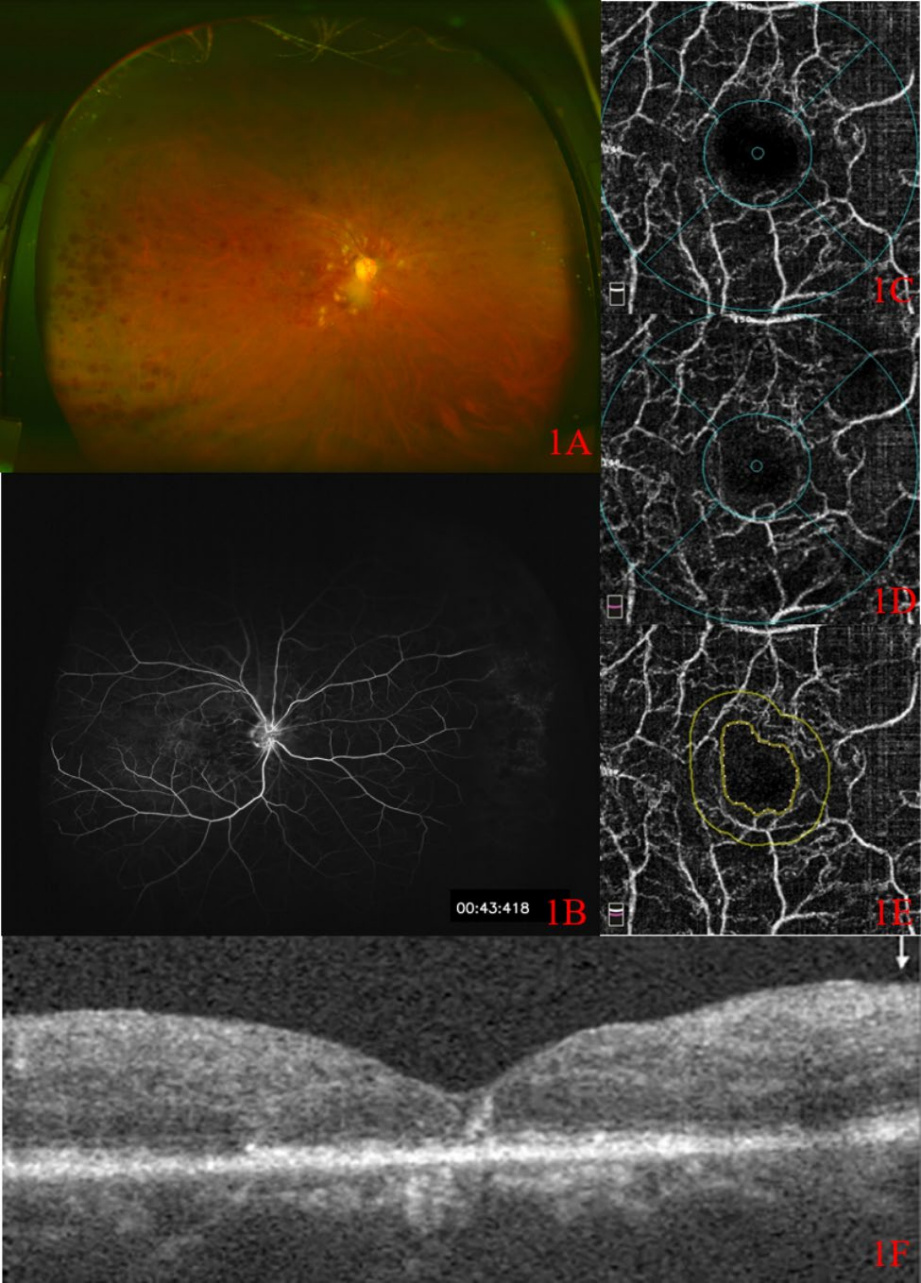
CFP, UWFFA and OCTA images of a patient with ischemic CRVO. 1 A: Widefield color fundus photograph; 1B: ultra-wide-angle fundus fluorescein angiography; 1 C: SCP in OCTA; 1D: DCP in OCTA; 1E: FAZ area in OCTA; 1 F: OCT

**Fig. 2:**
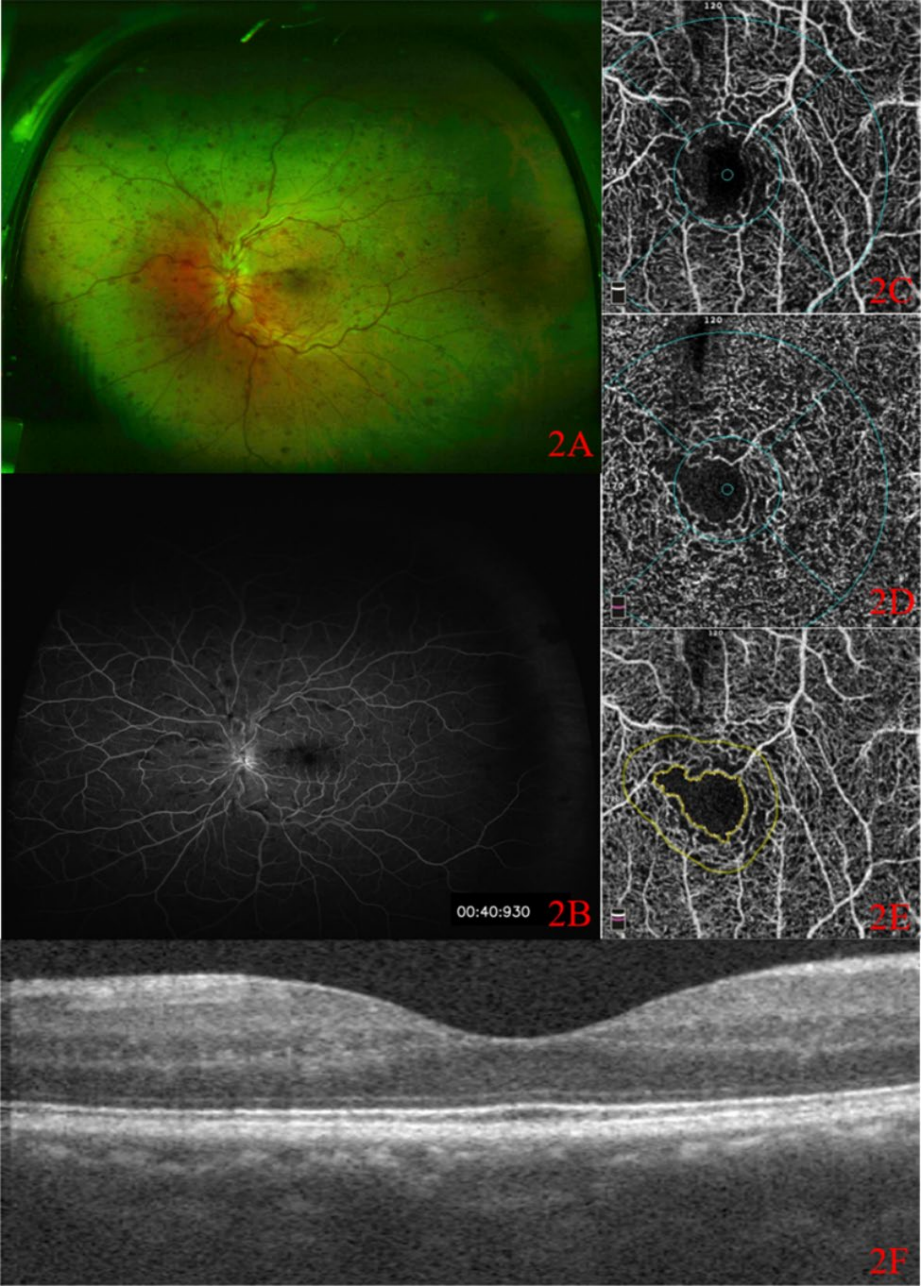
CFP, UWFFA and OCTA images of a patient with non-ischemic CRVO. 2 A: Widefield color fundus photograph; 2B: ultra-wide-angle fundus fluorescein angiography; 2 C: SCP in OCTA; 2D: DCP in OCTA; 2E: FAZ area in OCTA; 2 F: OCT

Ischemic group: Widefield color fundus photograph showed partial absorption of hemorrhage (Fig. 1 A), UWFFA showed large NP area (Fig. 1B), and OCTA revealed obvious loss of capillaries in SCP (Fig. 1 C) and DCP (Fig. 1D) and the damage of the capillary arcade (Fig. 1E). OCT showed a normal foveal contour with no cystoid macular edema. (Fig. 1 F). Non-ischemic group: Widefield color fundus photograph showed partial absorption of hemorrhage (Fig. 2 A). UWFFA showed relatively small NP area (Fig. 2B), while OCTA revealed loss of small capillaries in SCP (Fig. 2 C) and DCP (Fig. 2D) and the damage of the capillary arcade (Fig. 2E). OCT showed a normal foveal contour with no cystoid macular edema. (Fig. 2 F).

Analysis of OCTA data showed there were no significant differences in SFVD, DFVD and CRT between the ischemic and non-ischemic group (*P* > 0.05) and significant differences in SVD, SPFVD, DVD, DPFVD, FAZ area, PERIM, AI and FD-300 between the ischemic and non-ischemic group (*P* < 0.05) (Table 1).

**Table 1:** Comparison of OCTA parameters between ischemic and non-ischemic group

	BRVO (${\rm{\bar x}}\,{\rm{ \pm }}\,{\rm{s,}}$ %)		t	***P***
	Ischemic group	non-ischemic group		
**SVD**	36.58 ± 5.64	42.50 ± 4.25	-4.169	≤ 0.001^*^
**SFVD**	12.33 ± 4.62	11.73 ± 6.05	0.399	0.691
**SPFVD**	39.17 ± 6.28	45.10 ± 4.38	-3.841	≤ 0.001^*^
**DVD**	32.65 ± 4.73	43.98 ± 5.01	-8.219	≤ 0.001^*^
**DFVD**	20.54 ± 6.26	22.47 ± 7.23	-1.010	0.317
**DPFVD**	33.65 ± 5.12	45.73 ± 6.04	-7.647	≤ 0.001^*^
**CRT**	225.38 ± 39.49	234.33 ± 45.45	-0.745	0.460
**FAZ area**	0.81 ± 0.47	0.44 ± 0.16	3.672	0.001^*^
**PERIM**	4.39 ± 1.83	2.76 ± 0.64	4.125	≤ 0.001^*^
**AI**	1.32 ± 0.16	1.18 ± 0.09	3.550	0.001^*^
**FD-300**	38.27 ± 9.06	45.24 ± 5.10	-3.310	0.002^*^

*: *P* < 0.05 was considered statistically significant

**Fig. 3:**
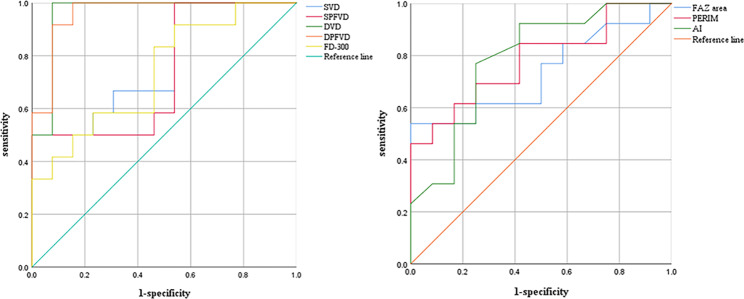
ROC curve on OCTA parameters in distinguishing ischemic from non-ischemic CRVO

ROC curve analysis of OCTA parameters with statistical significance between the two groups (SVD, SPFVD, DVD, DPFVD, FAZ area, PERIM, AI and FD-300) showed that the AUC of DVD and DPFVD in predicting ischemic CRVO was highest (0.926), and the threshold was all 38.40%. The sensitivity and specificity of DVD were 100% and 92.3%. The sensitivity and specificity of DPFVD were 100% and 84.6%, respectively. (Table 2; Fig. 3).

**Table 2:** ROC results for ischemic versus non-ischemic CRVO

	AUC	95%CI	
		**Lower**	**Upper**
**SVD**	0.776	0.647	0.905
**SPFVD**	0.737	0.595	0.880
**DVD**	0.962	0.907	1.000
**DPFVD**	0.962	0.913	1.000
**FD-300**	0.737	0.600	0.874
**FAZ area**	0.734	0.593	0.875
**PERIM**	0.782	0.655	0.909
**AI**	0.788	0.661	0.916

## Discussion

Previous studies have confirmed that there was a significant correlation between automatic quantification of macular VD and FAZ area by OCTA and the peripheral non-perfusion area measured by FFA for patients with RVO [14, 15]. However, there is no conclusive conclusion as to whether OCTA parameters can distinguish ischemic from non-ischemic CRVO and predict ischemic progression in patients with CRVO patients. Therefore, in this study, We used OCTA to quantitatively evaluate the differences in macular microvascular structure between ischemic and non-ischemic CRVO patients, and to analyze whether OCTA can differentiate ischemic and non-ischemic CRVO.

Macular edema is the most common complication of CRVO. Macular edema will interfere the automatic segmentation of SCP and DCP, affecting the automatic quantification of VD and the accurate analysis of OCTA image [15]. Kim et al. [16] divided RVO patients into two groups according to CRT, and researched the factors affecting the reproducibility of VD measured by OCTA. They found that the coefficient of variation of VD in the first group (CRT > 400 μm) was much greater than that of the second group (CRT < 400 μm), indicating the increase of CRT caused by macular edema would significantly affect the accuracy of VD measurement. Therefore, in this study, All patients were administered three consecutive intravitreal anti-VEGF injections until resolution of macular edema, which avoid errors caused by macular edema as much as possible and ensure the reliability of the data.

Adhi et al [17]. used OCTA to measure the FAZ area of normal people, which was 0.30 ± 0.09mm^2^, suggesting that the FAZ area of both groups in our study was larger than that of normal eyes. Xing et al [18]. used OCTA to measure the VD of normal people in the SCP and DCP, which were 49.00 ± 2.72 and 53.05 ± 3.26, respectively, suggesting the VD in the SCP and DCP were decreased in the two groups in this study. It is consistent with the results of previous studies. In addition, we found that compared with non-ischemic CRVO, the VD in SCP and DCP of ischemic CRVO was significantly decreased, and the FAZ area was obviously larger, indicating that the more severe the ischemia, the worse the perfusion of retinal vessels and the more serious the damage of the capillary arcade. Further analysis of OCTA parameters showed that SVD, SPFVD, DVD, DPFVD, FAZ area, PERIM, AI and FD-300 in ischemic group were significantly lower than those in non-ischemic group, indicating that the changes of these parameters were more closely related to severity of retinal ischemia. The more severe the ischemia, the greater reduction of these parameters.

In order to further find out the parameter most related to the degree of ischemia in CRVO, we performed ROC curve analysis on SVD, SPFVD, DVD, DPFVD, FAZ area, PERIM, AI and FD-300, and found that the AUC of DVD and DPFVD were highest, indicating that the VD reduction in DCP were most correlated with the degree of ischemia. The vascular structure of DCP that involves direct communication with major veins and the lack of vascular smooth muscles render the DCP most vulnerable to hemodynamic disturbances following CRVO and the ensuing hypoperfusion compared to the SCP [19]. In addition, SCP has less involvement and better perfusion owing to direct connection to retinal arterioles in contrast to DCP [15]. In this study, we found that the AUC of DVD and DPFVD in predicting ischemic CRVO was highest (0.962). the threshold was all 38.40%, and the sensitivity was all 100%, but the specificity of DVD (92.3%) was significantly higher than that of DPFVD (84.6%). Therefore, DVD ≤ 38.40% can be used as the best threshold for determining ischemic CRVO. It suggested that FFA is not necessary to judge the ischemic type for patients with newly diagnosed CRVO, and OCTA was recommended for closely follow-up during treatment. When DVD ≤ 38.40%, UWFFA should be performed to clarify the range of the retinal NP area and determine whether retinal laser photocoagulation is needed to prevent the occurrence of retinal neovascularization.

In conclusion, OCTA can accurately evaluate the changes of macular microvascular structure of the SCP and DCP, and quantitatively measure parameters such as VD and FAZ area, so as to accurately evaluate macular ischemia and predict the degree of peripheral retinal ischemia, which is conducive to the differentiation between ischemic and non-ischemic CRVO and the subsequent retinal laser photocoagulation treatment. Our study has several limitations. (1) This study was a cross-sectional study without a comparative observation before and after treatment. (2) the results are based on a relatively small sample size. (3) technical limitations including projection artifacts and motion artifacts that are inherent in the current technology might potentially affect the OCTA assessment. A future prospective, controlled study with a large sample size is needed to confirm the current results.

## Data Availability

The datasets generated and/or analysed during the current study are not publicly available due data do not have consent from all patients to share their information online but are available from the corresponding author on reasonable request.
